# Remote Ischemic Conditioning: A Novel Non-Invasive Approach to Prevent Post-Stroke Depression

**DOI:** 10.3389/fnagi.2017.00270

**Published:** 2017-08-08

**Authors:** Wenbo Zhao, Fang Jiang, Zhen Zhang, Jing Zhang, Yuchuan Ding, Xunming Ji

**Affiliations:** ^1^Department of Neurology, Xuanwu Hospital, Capital Medical University Beijing, China; ^2^Department of Neurosurgery, Wayne State University School of Medicine Detroit, MI, United States; ^3^Department of Neurosurgery, Xuanwu Hospital, Capital Medical University Beijing, China

**Keywords:** remote ischemic conditioning, post-stroke depression, stroke, neuropsychiatric complication, depression and anxiety disorders

## Abstract

Post-stroke depression (PSD) is a common neuropsychiatric complication of stroke. However, due to the high expense and side effects of pharmacotherapy and the difficult-to-achieve of psychotherapy, the prevention and treatment of PSD are still far from satisfaction. Inflammation hypothesis is now playing an essential role in the pathophysiological mechanism of PSD, and it may be a new preventive and therapeutic target. Remote ischemic conditioning (RIC) is a non-invasive and easy-to-use physical strategy, which has been used to protect brain (including ischemic and hemorrhagic stroke), heart and many other organs in clinical trials. The underlying mechanisms of RIC include anti-inflammation, anti-oxidative stress, immune system regulation and other potential pathways. Our hypothesis is that RIC is a novel approach to prevent PSD. The important implications of this hypothesis are that: (1) RIC could be widely used in clinical practice to prevent PSD if our hypothesis were verified; and (2) RIC would be thoroughly explored to test its effects on other neurobehavioral disorders (e.g., cognitive impairment).

## Introduction

Post-stroke depression (PSD) is one of the most frequent and important neuropsychiatric complications of stroke. One third of stroke survivors experienced major depression, and what’s worse, the prevalence of minor and moderate depression is much higher (Robinson and Jorge, 2016). Studies showed that PSD has adverse effects on functional recovery, cognitive function and social and interpersonal activities, and it can also increase mortality (10 times higher than in patients without PSD; Espárrago Llorca et al., 2015).

In clinical practice, however, PSD is generally undiagnosed and undertreated. Thus the prevention of PSD may be more important than its treatment in the real world. Psychotherapy and antidepressants (e.g., Escitalopram) may be effective in preventing the occurrence of PSD (Robinson et al., 2008; Nabavi et al., 2014). However, due to the high expense and side effects of pharmacotherapy and the difficult-to-achieve of psychotherapy, rigorous clinical trials are needed to determine their utility after acute stroke (Mohr et al., 2006; Peterson et al., 2017). Currently, the mechanisms of frequently-used antidepressants are largely based on the monoamine hypothesis, and all these pathways have anti-inflammatory effects directly or indirectly (Dwyer Hollender, 2014). Furthermore, inflammation hypothesis has been an important pathophysiological mechanism of depression (Kohler et al., 2016). Therefore, anti-inflammation may be a new target for the prevention and treatment of PSD.

Remote ischemic conditioning (RIC) is a protective systemic strategy by which one or more cycles of brief, nonlethal limb ischemia confer protection to distant organs (Meng et al., 2012; Hausenloy et al., 2015; Meybohm et al., 2015). It has been proven to be an effective strategy for cardioprotection in patients with ischemic cardiovascular diseases, and it is also effective for neuroprotection in patients with hemorrhagic stroke, acute ischemic stroke and chronic cerebral ischemia (Meng et al., 2012; Hougaard et al., 2014; Hausenloy and Yellon, 2016; Laiwalla et al., 2016; Zhao et al., 2017). The underlying mechanisms involved in providing RIC induced distant organs protection include anti-inflammation, anti-oxidative stress, immune system regulation, autonomous nervous system regulation and other potential pathways (Randhawa et al., 2015). Against these backgrounds, we assume that RIC may inhibit several pathways of PSD and have beneficial effects on the prevention of PSD.

## Theory of the Hypothesis

Our hypothesis is that RIC is a novel approach to prevent PSD. Currently, RIC has been widely used in clinical trial to test its effects on organic diseases of heart, brain, kidney, limb and other organs. It has been shown to inhibit recurrent stroke effectively in patients with ischemic stroke (Meng et al., 2012, 2015), reduce the incidence of new brain lesion on MRI after carotid artery stenting (Zhao et al., 2017), and improve functional outcomes in patients with hemorrhagic stroke (Laiwalla et al., 2016). However, no study focuses on RIC’s effects on psychological dysfunction.

Recently, several studies have provided support for the role of inflammatory response in the development of PSD, which has been further supported by clinical findings of increased serum inflammatory cytokines in patients who developed PSD (Spalletta et al., 2006, 2013; Li et al., 2014). Furthermore, inflammation has complex interactions with monoamine system, hypothalamic-pituitary-adrenal (HPA) axis and neuroplasticity, and all of them contribute to the pathophysiological mechanisms of PSD (Fang and Cheng, 2009; Li et al., 2014).

**Figure 1:**
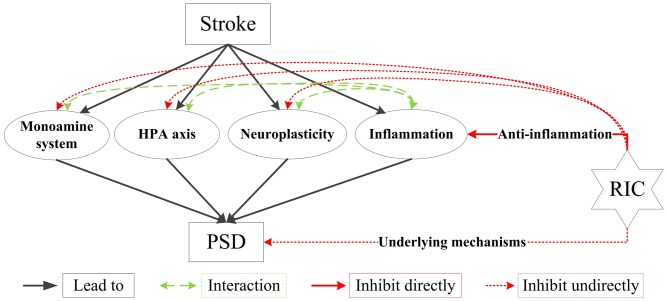
Main biological mechanisms of PSD and how RIC plays its roles in preventing PSD. HPA indicates hypothalamic-pituitary-adrenal; PSD, post-stroke depression; RIC, remote ischemic conditioning. Stroke leads to PSD through several biological mechanisms, such as the ascending monoamine systems, the abnormality of HPA axis, the alteration of neuroplasticity and inflammation. Inflammation also has complex interactions with monoamine systems, HPA axis and neuroplasticity. RIC can prevent PSD through anti-inflammation and other underlying mechanisms directly, and it has effects on monoamine systems, HPA axis and neuroplasticity indirectly. All these effects of RIC could be the potential pathways to prevent and treat PSD.

The mechanisms involved in providing RIC induced organs protection are quite complex and interlinked, but its effects on inflammation may be one of the most important ones (Hausenloy and Yellon, 2008; Randhawa et al., 2015). Clinical researches showed that RIC could reduce plasma inflammatory markers (e.g., high sensitive C-reactive protein, interleukin-6) in stroke patients (Meng et al., 2015). In addition, RIC has many other potential pathways to induce organ protection (Randhawa et al., 2015), and some of them may also exist in the underlying mechanism of PSD (Figure 1).

## Evaluation of the Hypothesis

Our hypothesis could be supported by the evidences that the mechanism of RIC includes anti-inflammation, immunoregulation, antioxidant and other underlying pathways, which are also existed in the pathophysiology of PSD.

Like many psychiatric disorders, however, psychological, social and biological factors all play their roles in the occurrence of PSD (De Ryck et al., 2014). Furthermore, PSD can be influenced by many other factors, including genetic factors, medical and psychiatric history, stroke characteristics and lesion location, and social support. Therefore, the effect of RIC on PSD might be limited in some kinds of patients, and selecting the potentially benefited populations may be a key issue. Therefore, ischemic stroke patients with left hemisphere infarction, greater severity and less social support might be better choice for clinical trials that determining the efficacy of RIC for PSD.

We plan to test our hypothesis by both animal experiment and clinical trial:

1. **Animal experiment**: stroke model will be induced by occluding the middle cerebral artery using the intraluminal filament technique, and then chronic mild stress will be applied to the model to induce depression (Willner et al., 1987). RIC will be applied to part of the stroke model. RIC will be initiated following the stroke by bilaterally occluding blood flow to the hind limbs with method used by Ren et al. (2015). The incidence of PSD and the concentration of monoamine neurotransmitter in brain tissue will be recorded to measure the results.
2. **Clinical trial**: we will recruit ischemic stroke patients and apply RIC to them by the method used by Meng et al. (2012), and RIC will be performed twice daily for 6 months. The Diagnostic and Statistical Manual of Mental Disorders, Fifth Edition (DSM-5) will be used to determine depression, Hamilton Depression Rating Scale (HAMD) will be used to evaluate the severity of depression, and plasma biomarkers (including interleukin 1, interleukin 6 and interleukin 10) will be tested to determine the underlying mechanisms. The primary outcome is the incidence of PSD within 6 months after stroke onset. The secondary outcomes include: (1) recovery of neurological dysfunction; (2) change in plasma biomarkers; (3) recurrent of ischemic cerebrovascular events; and (4) any adverse events.

## Important Implications of the Hypothesis

If this hypothesis were verified by animal and clinical studies, RIC could be widely used in clinical practice to prevent PSD and thoroughly explored to test its effects on other neurobehavioral disorders (e.g., cognitive impairment). Compared with current strategies (i.e., psychotherapy and pharmacotherapy) for the treatment of PSD and other neurobehavioral disorders, the advantages of RIC include that: (1) it is a non-expensive and safe therapy, and no severe side effects has been reported; (2) it has multiple organs (e.g., brain, hear, kidney, liver, limbs, lung) protection, and multiple diseases preventions and treatment; (3) its usage can be learnt easily, and it is easily to be used, which even can be done with a sphygmomanometer; and (4) it can be done without the requirement of special place. Of course, RIC still has several limitations, which may limit its popularization. To date, we still do not know the exact mechanisms and the optimal protocol of RIC, and it may be time consume and cause skin petechiae. In addition, RIC cannot be performed on limbs with any vascular, soft tissue, or orthopedic injury (Zhao et al., 2017).

## Conclusion

RIC might be a novel non-invasive and easy-to-use strategy for preventing PSD. Functional recovery of stroke survivors would be further improved and the mortality would be decreased significantly. If our hypothesis is confirmed by our animal experiment and clinical trial, RIC would be thoroughly explored in clinical trials to test its effects on psychological disorders and other post stroke neurobehavioral disorders (e.g., cognitive impairment).

## Author Contributions

WZ and FJ wrote the draft. WZ made the Figure. All authors contributed to the critical revision of the manuscript and have approved the final version of this review article.

## Conflict of Interest Statement

The authors declare that the research was conducted in the absence of any commercial or financial relationships that could be construed as a potential conflict of interest.

## References

[B1] De Ryck A. Fransen E. Brouns R. Geurden M. Peij D. Mariën P.. (2014). Poststroke depression and its multifactorial nature: results from a prospective longitudinal study. J. Neurol. Sci. 347, 159–166. 10.1016/j.jns.2014.09.038 25451004

[B2] Dwyer Hollender K. (2014). Screening, diagnosis, and treatment of post-stroke depression. J. Neurosci. Nurs. 46, 135–141. 10.1097/JNN.0000000000000047 24670433

[B3] Espárrago Llorca G. Castilla-Guerra L. Fernández Moreno M. C. Ruiz Doblado S. Jiménez Hernández M. D. (2015). Post-stroke depression: an update. Neurologia 30, 23–31. 10.1016/j.nrl.2012.06.008 22901370

[B4] Fang J. Cheng Q. (2009). Etiological mechanisms of post-stroke depression: a review. Neurol. Res. 31, 904–909. 10.1179/174313209X385752 19891854

[B7] Hausenloy D. J. Candilio L. Evans R. Ariti C. Jenkins D. P. Kolvekar S.. (2015). Remote ischemic preconditioning and outcomes of cardiac surgery. N. Engl. J. Med. 373, 1408–1417. 10.1056/NEJMoa1413534 26436207

[B5] Hausenloy D. J. Yellon D. M. (2008). Remote ischaemic preconditioning: underlying mechanisms and clinical application. Cardiovasc. Res. 79, 377–386. 10.1093/cvr/cvn114 18456674

[B6] Hausenloy D. J. Yellon D. M. (2016). Ischaemic conditioning and reperfusion injury. Nat. Rev. Cardiol. 13, 193–209. 10.1038/nrcardio.2016.5 26843289

[B8] Hougaard K. D. Hjort N. Zeidler D. Sørensen L. Nørgaard A. Hansen T. M.. (2014). Remote ischemic perconditioning as an adjunct therapy to thrombolysis in patients with acute ischemic stroke: a randomized trial. Stroke 45, 159–167. 10.1161/STROKEAHA.113.001346 24203849

[B9] Kohler O. Krogh J. Mors O. Benros M. E. (2016). Inflammation in depression and the potential for anti-inflammatory treatment. Curr. Neuropharmacol. 14, 732–742. 10.2174/1570159x14666151208113700 27640518 PMC5050394

[B10] Laiwalla A. N. Ooi Y. C. Liou R. Gonzalez N. R. (2016). Matched cohort analysis of the effects of limb remote ischemic conditioning in patients with aneurysmal subarachnoid hemorrhage. Transl. Stroke Res. 7, 42–48. 10.1007/s12975-015-0437-3 26630942 PMC4724226

[B11] Li W. Ling S. Yang Y. Hu Z. Davies H. Fang M. (2014). Systematic hypothesis for post-stroke depression caused inflammation and neurotransmission and resultant on possible treatments. Neuro Endocrinol. Lett. 35, 104–109. 24878979

[B12] Meng R. Asmaro K. Meng L. Liu Y. Ma C. Xi C.. (2012). Upper limb ischemic preconditioning prevents recurrent stroke in intracranial arterial stenosis. Neurology 79, 1853–1861. 10.1212/WNL.0b013e318271f76a 23035060

[B13] Meng R. Ding Y. Asmaro K. Brogan D. Meng L. Sui M.. (2015). Ischemic conditioning is safe and effective for octo- and nonagenarians in stroke prevention and treatment. Neurotherapeutics 12, 667–677. 10.1007/s13311-015-0358-6 25956401 PMC4489956

[B14] Meybohm P. Bein B. Brosteanu O. Cremer J. Gruenewald M. Stoppe C.. (2015). A multicenter trial of remote ischemic preconditioning for heart surgery. N. Engl. J. Med. 373, 1397–1407. 10.1056/NEJMoa1413579 26436208

[B15] Mohr D. C. Hart S. L. Howard I. Julian L. Vella L. Catledge C.. (2006). Barriers to psychotherapy among depressed and nondepressed primary care patients. Ann. Behav. Med. 32, 254–258. 10.1207/s15324796abm3203_12 17107299

[B16] Nabavi S. F. Turner A. Dean O. Sureda A. Mohammad S. (2014). Post-stroke depression therapy: where are we now? Curr. Neurovasc. Res. 11, 279–289. 10.2174/1567202611666140522123504 24852795

[B17] Peterson K. Dieperink E. Anderson J. Boundy E. Ferguson L. Helfand M. (2017). Rapid evidence review of the comparative effectiveness, harms, and cost-effectiveness of pharmacogenomics-guided antidepressant treatment versus usual care for major depressive disorder. Psychopharmacology 234, 1649–1661. 10.1007/s00213-017-4622-9 28456840

[B18] Randhawa P. K. Bali A. Jaggi A. S. (2015). RIPC for multiorgan salvage in clinical settings: evolution of concept, evidences and mechanisms. Eur. J. Pharmacol. 746, 317–332. 10.1016/j.ejphar.2014.08.016 25176179

[B19] Ren C. H. Wang P. C. Wang B. Li N. Li W. G. Zhang C. G.. (2015). Limb remote ischemic per-conditioning in combination with post-conditioning reduces brain damage and promotes neuroglobin expression in the rat brain after ischemic stroke. Restor. Neurol. Neurosci. 33, 369–379. 10.3233/RNN-140413 25868435 PMC4923706

[B20] Robinson R. G. Jorge R. E. (2016). Post-stroke depression: a review. Am. J. Psychiatry 173, 221–231. 10.1176/appi.ajp.2015.15030363 26684921

[B21] Robinson R. G. Jorge R. E. Moser D. J. Acion L. Solodkin A. Small S. L.. (2008). Escitalopram and problem-solving therapy for prevention of poststroke depression: a randomized controlled trial. JAMA 299, 2391–2400. 10.1001/jama.299.20.2391 18505948 PMC2743160

[B22] Spalletta G. Bossù P. Ciaramella A. Bria P. Caltagirone C. Robinson R. G. (2006). The etiology of poststroke depression: a review of the literature and a new hypothesis involving inflammatory cytokines. Mol. Psychiatry 11, 984–991. 10.1038/sj.mp.4001879 16894392

[B23] Spalletta G. Cravello L. Imperiale F. Salani F. Bossù P. Picchetto L.. (2013). Neuropsychiatric symptoms and interleukin-6 serum levels in acute stroke. J. Neuropsychiatry Clin. Neurosci. 25, 255–263. 10.1176/appi.neuropsych.12120399 24247852

[B24] Willner P. Towell A. Sampson D. Sophokleous S. Muscat R. (1987). Reduction of sucrose preference by chronic unpredictable mild stress, and its restoration by a tricyclic antidepressant. Psychopharmacology 93, 358–364. 10.1007/bf00187257 3124165

[B25] Zhao W. Meng R. Ma C. Hou B. Jiao L. Zhu F.. (2017). Safety and efficacy of remote ischemic preconditioning in patients with severe carotid artery stenosis before carotid artery stenting: a proof-of-concept, randomized controlled trial. Circulation 135, 1325–1335. 10.1161/CIRCULATIONAHA.116.024807 28174194 PMC5802341

